# The Role of cGAS-STING Signalling in Metabolic Diseases: from Signalling Networks to Targeted Intervention

**DOI:** 10.7150/ijbs.84890

**Published:** 2024-01-01

**Authors:** Jiahui Gong, Xilong Gao, Shaoyang Ge, Hongliang Li, Ran Wang, Liang Zhao

**Affiliations:** 1 College of Food Science and Nutritional Engineering, China Agricultural University, Beijing 100083, China.; 2 Key Laboratory of Functional Dairy, Department of Nutrition and Health, China Agricultural University, Beijing 100193, China.; 3 Hebei Engineering Research Center of Animal Product, Sanhe 065200, China.; 4 Inner Mongolia Mengniu Dairy (Group) Co., Ltd., Hohhot 011517, China.; 5 Research Center for Probiotics, China Agricultural University, Sanhe 065200, China.; 6 Food Laboratory of Zhongyuan, Luohe 462300, China.

**Keywords:** cGAS, STING, metabolic diseases, inflammation, signalling network, target intervention

## Abstract

The cyclic GMP-AMP synthase (cGAS)-stimulator of interferon genes (STING) is a crucial innate defence mechanism against viral infection in the innate immune system, as it principally induces the production of type I interferons. Immune responses and metabolic control are inextricably linked, and chronic low-grade inflammation promotes the development of metabolic diseases. The cGAS-STING pathway activated by double-stranded DNA (dsDNA), cyclic dinucleotides (CDNs), endoplasmic reticulum stress (ER stress), mitochondrial stress, and energy imbalance in metabolic cells and immune cells triggers proinflammatory responses and metabolic disorders. Abnormal overactivation of the pathway is closely associated with metabolic diseases such as obesity, nonalcoholic fatty liver disease (NAFLD), insulin resistance and cardiovascular diseases (CVDs). The interaction of cGAS-STING with other pathways, such as the nuclear factor-kappa B (NF-κB), Jun N-terminal kinase (JNK), AMP-activated protein kinase (AMPK), mammalian target of rapamycin (mTOR), autophagy, pyroptosis and insulin signalling pathways, is considered an important mechanism by which cGAS-STING regulates inflammation and metabolism. This review focuses on the link between immune responses related to the cGAS-STING pathway and metabolic diseases and cGAS-STING interaction with other pathways for mediating signal input and affecting output. Moreover, potential inhibitors of the cGAS-STING pathway and therapeutic prospects against metabolic diseases are discussed. This review provides a comprehensive perspective on the involvement of STING in immune-related metabolic diseases.

## Introduction

Innate immunity is a natural defence barrier in our body that protects host cells from foreign pathogens recognized by pattern recognition receptors (PRRs). The discovery in 2013 of the cytoplasmic DNA sensor cyclic GMP-AMP synthase (cGAS), a novel intracellular signal transduction-related PRR, drives the production of stimulator of interferon genes (STING) proteins 1. Since its discovery, STING has received widespread attention because of its pleiotropic functions 2. Notably, the cGAS-STING pathway is crucial for hosts defending against infection by viruses, microbial pathogens, and other infections via innate immunity. cGAS also contributes to bacterial resistance against phages, underscoring its evolutionary conservation 3. Further research into the cGAS pathway has revealed that in addition to exogenous DNA exposure, endogenous DNA damage can self-DNA molecules to be located within the cytoplasmic matrix. The presence of DNA in cytoplasm can trigger STING-mediated innate immune responses, as well as adaptive immune responses. In contrast to its well-defined role in innate immunity, STING pathway function in adaptive immunity appears to be largely unregulated manner in various tissues and cell types. The persistent abnormal modulation of the cGAS-STING pathway causes perpetual activation of the body's immune system, resulting in reduced of immune tolerance, increased tissue and organ damage, and even biosystem dysfunction. Thus, the resulting chronic low-level inflammation within the body may ultimately lead to the development of autoimmune diseases 4, metabolic diseases 5, respiratory diseases 6, neurodegenerative diseases 7, and tumours 8.

The global epidemics of common and highly prevalent metabolic diseases such as obesity, Type 2 diabetes mellitus (T2DM), nonalcoholic fatty liver disease (NAFLD) and cardiovascular diseases (CVDs) are serious threats to human health and constitute a major public health burden 9, 10. Healthy metabolism is essential for the energy intake and utilization by the body as well as the surveillance functions of the immune system 11. When one or more pathways and functions related to nutrient perception, as well as the detection of pathogens and related immune responses, are disrupted, and metabolism is affected, possibly resulting in the development of metabolic diseases 11. In general, metabolic diseases are influenced by genetic and environmental factors, with obesity caused by overnutrition being a major trigger 10. According to the Centers for Disease Control and Prevention (CDC) statistics, the prevalence of obesity among adults in the United States will be as high as 33.0% in 2021^12^. Overeating can cause multisystem metabolic disorders such as inflammatory responses, insulin resistance, mitochondrial dysfunction, and ER stress due to the ectopic accumulation of fat and imbalanced glucolipid metabolism 13. Obesity is closely associated with a variety of metabolic diseases, such as T2DM, CVD, NAFLD. Notably, the concept of 'meta-inflammation' has been introduced to explain the interconnectedness of nutrient utilization and the regulation of metabolic homeostasis. This term underscores the idea that chronic low-level inflammation connects obesity to metabolic diseases 14. The metabolic activity and immune response of the body are energy dependent. When energy metabolism is imbalanced, varying levels of inflammatory factors, chemokines released, oxidative stress and metabolic dysfunction characterize specific metabolic tissues and immune cells. These impairments and inflammatory mediators subject the immune system to significant stress and disrupt the normal metabolic functions of adipose tissue, liver, skeletal muscle, vascular endothelium, and other bodily components. Ultimately, this disruption leads to the development of metabolic diseases 13. Therefore, in-depth exploration into the regulatory role of innate and adaptive immunity in metabolic diseases may help in developing strategies to reverse the progression of metabolic diseases from an immunological perspective.

In recent years, the cGAS-STING pathway has garnered increasing recognition as a potent regulator of chronic inflammatory and metabolic diseases and cancer. In this review, we aim to characterize into the classical mechanisms and dual roles of the STING pathway in innate immunity. Subsequently, we shed light on the pivotal roles played by the STING pathway in metabolic diseases such as obesity, NAFLD, insulin resistance, and cardiovascular diseases. We reveal its multifaceted functions in various cell types, including adipocytes, immune cells, endothelial cells, and hepatocytes, and within different tissues, such as adipose tissue, the liver, the pancreas, and the heart. Furthermore, we integrate its closely related upstream regulators, downstream effectors, and factors involved in its intricate cross-talk with other signalling pathways. Finally, we present a comprehensive summary and provide an update on recent advances in the research on STING-targeted inhibitors and potential interventions for metabolic diseases. Our purpose is to illuminate the profound implications of the cGAS-STING pathway on metabolic equilibrium preservation and to suggest novel targets of intervention in the context of metabolic diseases.

## The cGAS-STING pathway of cytosolic DNA sensing

The cGAS-STING pathway is dominated by STING (also known as MITA, MPYS, ERIS or TMEM173), a key regulator discovered in 2008 2. It is a conserved protein found in various vertebrates and is expressed at various levels in different tissues 15, with higher expression observed in the spleen, pancreas, and lymph nodes in rats 16 and in the lung, spleen, bone marrow, and appendix in humans 17. Single nucleotide polymorphisms in STING can affect its activity and innate immune responses in humans and bats 18.

**Figure 1:**
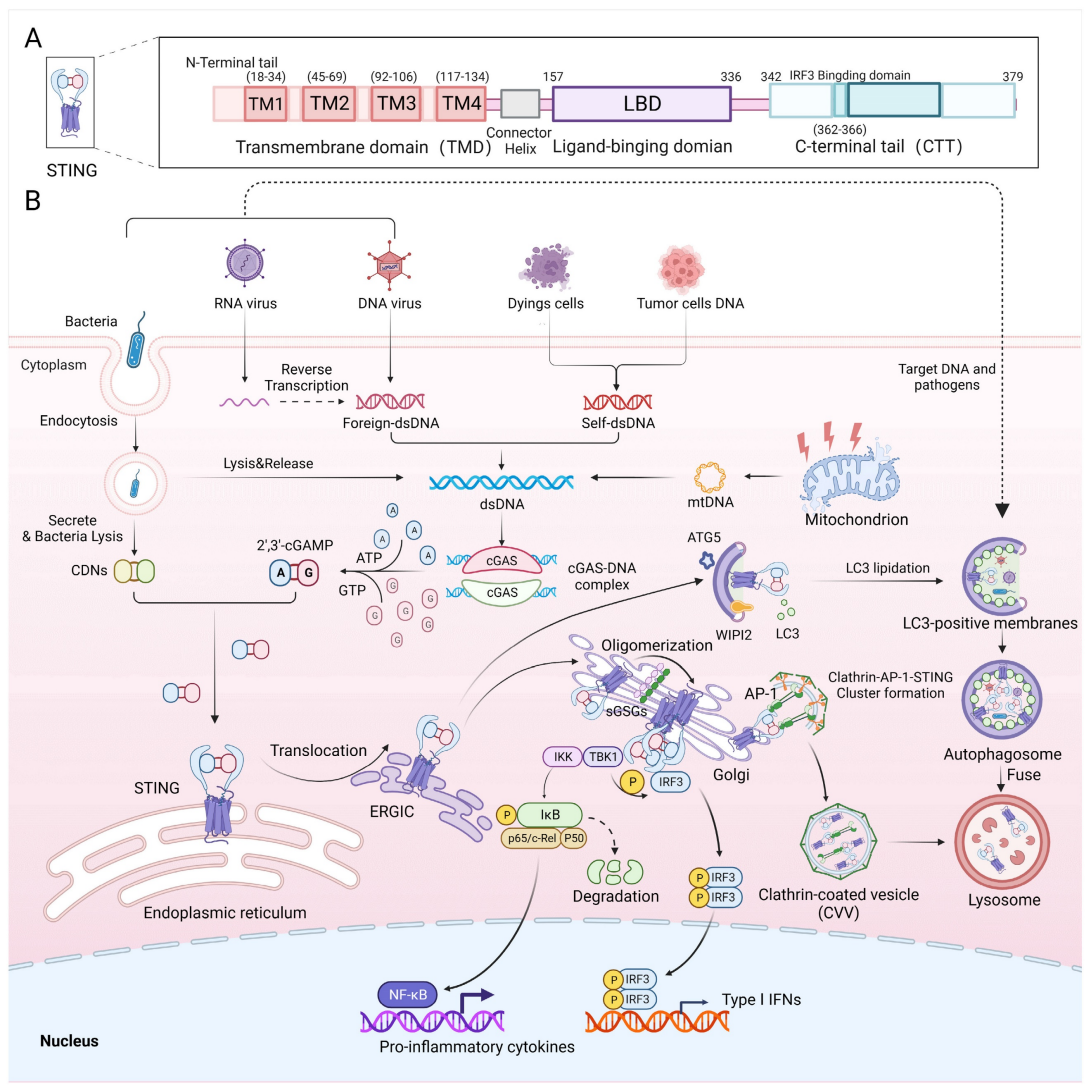
** cGAS-STING signalling in innate immunity. A)** Schematic diagram showing the functional structural domains of human STING. **B)** Double-stranded DNA from viruses, cancer cells and dead cells can activate the DNA sensor cGAS and form the cGAS-DNA complex, which then synthesizes cGAMP using ATP and GTP. Both CDNs from bacteria and cGAMP can bind to STING at the ER, causing STING to translocate to the ERGIC and then to the Golgi. Sulfated glycosaminoglycans (sGAGs) in the Golgi promote STING oligomerization and contribute to STING recruitment of TBK1. Autophosphorylation of TBK1 promotes the phosphorylation and dimerization of IRF3 and activates the nuclear transcription factor NF-κB. IRF3 and NF-κB entry into the nucleus mediates the production of proinflammatory cytokines and Type I interferons, which exert antiviral effects. Additionally, activated STING can form a clathrin-AP-1-STING cluster, which advances the development of CCVs, contributing to the degradation of STING in lysosomes. The STING-containing ERGIC acts as a membrane source for LC3 lipidation and induces LC3 lipidation via WIPI3 and ATG2, which induces autophagosome formation. LC3 on the membrane recruits DNA and pathogens to autophagosomes, which subsequently merge with lysosomes to promote pathogen degradation.

As an essential component of the innate immune system, the cGAS-STING pathway defends against DNA-containing pathogens or self-DNA by producing Type I interferons and inflammatory cytokines 19 (Figure 1B). The canonical cGAS-STING pathway includes cGAS, STING, TANK binding kinase 1 (TBK1) and interferon regulatory factor 3 (IRF3). Cytoplasmic double-stranded DNA from microbial infection (bacterial DNA, viral DNA or reverse transcribed viral RNA), mitochondria, tumour cells or dead cells binds to and activates cyclic GMP-AMP synthase (cGAS) 1, which starts a signalling cascade reaction. Activated cGAS uses ATP and GTP as substrates to catalyse the synthesis of 2'3'-cGAMP, which acts as a second messenger. Subsequently, cGAMP binds to the endoplasmic reticulum (ER) membrane adaptor STING to change its conformation and induce its activation. Then, STING is trafficked from the ER to an ER-Golgi intermediate compartment (ERGIC) and then to the Golgi apparatus, where it recruits and activates TBK1 via the STING TBK1-binding motif (PLPLRT/SD) located in the C-terminal tail (CTT) 20. Phosphorylated TBK1, in turn, phosphorylates the Ser366 site in the CTT domain of STING, allowing STING to recruit IRF3 via the pLxIS (p, hydrophilic residue; x, any residue; S, phosphorylation site) motif 21. Phosphorylated IRF3 dimerizes and then enters the nucleus. STING also activates IkB kinase (IKK), which phosphorylates IkB and in turn activates nuclear factor-kappa B (NF-κB). Then, NF-κB enters the nucleus, where it functions together with IRF3 and other transcription factors to induce the expression of Type I interferons and inflammatory cytokines such as tumour necrosis factor-alpha (TNF-α), interleukin 1β (IL-1β) and IL-6 22 (Figure 1B). Activated STING is transported to lysosomes via clathrin-coated vesicles (CCVs), where AP-1 can be recognized by a highly conserved acidic dileucine-based consensus motif ([D/E]XXXL[L/I]) in the C-terminal end of STING, inducing AP-1 binding to STING for transferring STING to CCVs 23. This process is also dependent on the TBK1 phosphorylation of STING. cGAS-STING is not only responsible for pathogen defence but is also involved in defending against autoimmune diseases and cancer 4, 8. Recently, studies showed that STING, as well as signalling factors upstream and downstream of it, can regulate inflammation and metabolism pathways.

In recent years, more details of the STING structure and mechanism of action have been elucidated. STING is an endoplasmic reticulum membrane protein consisting of 379 amino acids with a molecular weight of 42 kDa. It consists of an N-terminal tail; four transmembrane domains (TMDs), namely, transmembrane (TM1) (18-34), TM2 (45-69), TM3 (92-106) and TM4 (117-134); a ligand-binding domain (LBD); and a CTT 24 (Figure 1A). The LBD is responsible for binding to cGAMP, which activates STING, and the CTT contains the site of phosphorylation by TBK1 and the binding site for IRF3. Recently, exogenous activator Compound 53 (C53) was used to identify binding sites in the TMD of human STING that promote conformational changes in STING 25. These sites constitute the mechanism of STING oligomerization, and their discovery facilitates the development of a suitable secondary agonist or can be used as a binding site for novel STING agonists. Additionally, the mechanism of C53 binding is highly analogous to that of sulfated glycosaminoglycans (sGAGs) found in the Golgi, which also affect STING activation by binding to positively charged amino acid residues in the TMD domain of STING 26 (Figure 1B).

## The upstream signalling molecules that trigger STING activation

### Cytosolic DNA

As an important immune pathway for DNA sensing, the cGAS-STING pathway is naturally activated by cytosolic double-stranded DNA 1. Cytosolic DNA from the nucleus, mitochondria, viruses or bacteria can bind to cGAS and stimulate the cGAS-STING pathway, triggering immune responses 22. In adipocytes, transfection of synthetic viral DNA (HSV60) activated cGAS-STING and regulated the differentiation, proliferation and expression of adipocytokines, in which p204, not cGAS, acted as a cytosolic DNA sensor 27. Cytosolic mtDNA stimulation of the cGAS-STING pathway was observed in adipocytes 28, the endothelium 29, 30 and cardiomyocytes 31, thereby regulating inflammation, macrophage infiltration, insulin resistance, atherosclerosis and obesity-induced diabetic cardiomyopathy. The dysfunction of mitochondria induced by a HFD or palmitic acid is considered a trigger of mtDNA release 28.

### Cytosolic nuclease

Cytosolic nucleases are responsible for the degradation of cytosolic DNA and relieve the activation of cGAS-STING. Knockout nucleases, such as TREX1 (DNAse III), DNAse II and RNaseH2, lead to activation of cGAS-STING, subsequently causing autoimmune responses or lethality in a STING-dependent manner 4. Trex1 knockout mice exhibited systemic inflammation due to the activation of the cGAS-STING pathway. In addition, Trex1 knockout reduced adiposity and increased energy expenditure, which reduced mTORC1 activity, but not IRF3 activity, through cGAS-STING-TBK1 pathway-induced systemic metabolic defects 32. Repression of DNase II or Trex1 caused cytoplasmic DNA accumulation and activation of the TBK1-IRF3-IFN-β pathway. Reduced expression of DNase II and Trex1 was observed in HSCs from HFD-fed obese mice, and this effect was accompanied by the expression of senescence markers, particularly the expression of SASP factors, in HSCs and the development of hepatocellular carcinoma (HCC) 33. These results suggest that the ability of different types of nucleases to maintain the length, concentration, activity and stability of intracellular dsDNA is influenced by the environment in which the cell is located and may therefore lead to metabolic responses with different outcomes.

### cGAMP

Mammalian endogenous cGAMP is a 2'-3'-cGAMP formed via a 2'-5' phosphodiester bond and a 3'-5' phosphodiester bond, and it is synthesized by intracellular cGAS, which uses using ATP and GTP to bind and activate STING located in the endoplasmic reticulum 1, 22. Interestingly, exogenous cGAMP plays a different role in STING activation, especially in different cell types 34. Exogenous cGAMP aggravated inflammation in BMDMs but reduced proinflammatory responses in primary hepatocytes and 3T3-L1 adipocyte cells, and in the latter case, the anti-inflammatory effects different from those observed after STING activation in hepatocytes and adipocytes 34. Exogenous cGAMP suppressed gluconeogenesis and fat deposition in hepatocytes and increased insulin sensitivity of adipocytes, and both these effects were dependent on AKT 34. Intraperitoneally exogenous cGAMP decreased the strength of proinflammatory responses, attenuated metabolic dysregulation and led to glucose homeostasis in both the liver and adipose tissue of HFD-fed mice while decreasing liver TBK1 phosphorylation 34. Exogenous cGAMP may alleviate HFD-induced metabolic disorders, but more evidence is needed to explain the tissue-specific effects of exogenous cGAMP.

### Cyclic dinucleotide (CDN) and CDN analogues

In addition to 2′,3′-cGAMP, as mentioned above, 3′,3′-cGAMP, 3′,3′-di-AMP (cdA) and 3′,3′-di-GMP (cdG) produced by some bacteria can enter host cell to directly activate STING 35. However, the different single nucleotide polymorphisms (SNPs) in the human STING (hSTING) gene and the limited potency of natural CDNs reduce the broad-spectrum activation of STING by CDNs. To overcome this inhibitory effect on hSTING, synthetic CDN agonists have been developed by modifying their structure to increase their stability, potency and target specificity 36. The efficacy of exogenous CDN depends on the carrier used for cell entry; for example, the reduced-folate transporter SLC19A1 is expressed in human cells but not in mouse cells 37, 38. The antiviral efficacy of exogenous CDN may vary between species and tissues and depends on the transport proteins available in the respective tissues. For example, an exogenous CDN (c-di-GMP) can resist transoral infection with Drosophila C virus (DCV) and syndromic virus (SINV) via the action of the apical nucleoside transporter CNT2 in intestinal epithelial cells but acts only against systemically infection with DCV but not with SINV 39.

### Lipopolysaccharide (LPS)

LPS is a cell wall component of gram-negative bacteria. LPS is a ligand of TLR4 and induces cellular proinflammatory signalling through the TLR4-MyD88-IKKα/β/γ-NF-κB or TLR4-TRIF-TBK1-IRF3 pathway 40. Diet-induced dyshomeostasis of gut bacteria and an impaired gut barrier promote LPS translocation from the gut to the circulatory system and cause chronic proinflammation, which is considered an important factor in the development of obesity and insulin resistance 41. LPS induces signalling upstream of IRF3 and can stimulate IFN-β expression in macrophages 42. IRF3 was needed for LPS-induced insulin resistance in cultured 3T3-L1 adipocytes 43. LPS reduced the expression of STING in a human leukaemia plasmacytoid dendritic cell line (PMDC05 cells) 44. To date, no evidence indicates an interaction between LPS and TBK1.

### Agonists

The instability of cyclic dinucleotides and their analogues limits their use in immunotherapy. In addition, a large number of nonnucleotide STING agonists have been discovered; they include some flavonoid compounds, DMXAA and 10-carboxymethyl-9-acridanone (CMA), which can bind and activate mouse STING but not human STING, with binding to STING similar to that of cGAMP 45. As a cGAMP analogue, DMXAA promoted LPS-induced proinflammatory signalling in macrophages, hepatocytes and adipocytes. In recent years, research on STING agonists has been increasingly focused on how to achieve targeted delivery and increase the immune response to solid tumours. Commonly, nanocarriers such as liposomes, polymer micelles, polymer bodies and other inorganic nanoparticles are used to solve the problem of STING agonists not being able to easily penetrate various biological barriers *in vivo* and having low bioavailability 46. A nanoagonist based on bovine serum albumin (BSA)/ferritin combined with manganese (II) and β-lapachone has been shown to target the tumour microenvironment (TME) and dissociate to release Mn^2+^, which targets DC cells, to enhance the dsDNA binding capacity of cGAS, and β-lapachone, which targets tumour cells to induce apoptosis, thus initiating signalling cascades that increase local antitumour effects 47.

## The downstream signalling mediated via STING activation

### TBK1-related signalling molecules

TBK1 consists of a kinase domain (KD) located at the N-terminal end, a ubiquitin-like domain (ULD) and an α-helical scaffold dimerization domain (SDD) 48, and among these regions, the KD is essential for the activation of downstream substances. p62/sequestosome 1 (SQSTM1) is an effector of TBK1. Activated TBK1 phosphorylates p62, promoting lipotoxicity-induced p62 aggregates in hepatocytes and leading to NASH. TBK1 also induces ubiquitinated STING to undergo autophagosome-related degradation by phosphorylating p62/SQSTM1 49. The phosphorylation mutant Rab7-S72 (with the mutation at the S72 site) of the Ras-related protein Rab-7a (Rab7), a recently identified substrate of TBK1/IKKε, prevents the degradation of STING in lysosomes 50. In addition, in high-fat diet-induced obesity, overexpression of TBK1 and IKKε leads to phosphorylation of PDE3B, thereby reducing catecholamine-induced uncoupling protein 1 (UCP1)-mediated oxidative phosphorylation activation, which decreases energy expenditure; it also affects hormone-sensitive lipase activity via the PDE3B-cAMP-PKA pathway, reducing lipolysis and exacerbating energy imbalance 51. In β-cells, TBK1 can also mediate the cAMP-cAMP-dependent PKA-mammalian target of the rapamycin (mTOR) signalling pathway to prevent the replication and regeneration of β-cells 52. TBK1 regulates the survival of lung cancer cells carrying oncogenic KRAS mutations by phosphorylating the mitogenic kinase Polo-like kinase 1 (PLK1) 53. The interaction of TBK1 with NF-κB or JNK leads to the activation of NF-κB or JNK, which is responsible for the secretion of inflammatory cytokines 2, 54. These cytokines result in amplified inflammation-related signalling cascades and aggravated obesity-related metabolic disease.

### IRF3-related signalling molecules

The transcription factor IRF3 is a canonical effector molecule downstream of cGAS-STING. It is phosphorylated directly by TBK1 and enters the nucleus, regulating the expression of type I interferon (IFN-α/β) 21, 40. IFN-α/β can bind to IFNAR1 or IFNAR2 and induce the gene encoding cGAS. This positive feedback mechanism amplifies the activation of cGAS-STING 55. As a transcription factor, IRF3 has also been observed to bind to the promoters of other proteins and regulate their expression; it is considered a downstream signalling molecule of the STING-IRF3 pathway in metabolic disease. Activated IRF3 directly bound to the ICAM-1 promoter and induced ICAM-1 expression in a STING-dependent manner in endothelial cells. In this process, ICAM-1 acted as an effector of cGAS-STING signalling in diet-induced monocyte-endothelial cell adhesion and endothelium inflammation 29, 56. MST1 was the effector of STING-IRF3 in palmitic acid (PA)-induced inhibition of endothelial angiogenesis, in which phosphorylated IRF3 increased MST1 expression by binding to its promoter and induced YAP inactivation and inhibited angiogenesis 30. SCD1 was suppressed by IRF3 binding to its promoter in a TRIF-dependent manner 57. MCP-1 (CCL2) was also regulated by IRF3 via the TLR4/TRIF/IRF3 signalling cascade in palmitate/TNF-α-treated human monocytes 58.

## The cGAS-STING pathway in innate immunity

The cGAS-STING signalling pathway provides an important line of defence for the host against pathogen invasion and triggers immune defence. DNA from viruses or bacteria and cytoplasmic self-DNA are the main substrates of cytoplasmic sensors (cGASs). cGAS recognizes not only DNA viruses such as Kaposi's sarcoma-associated herpesvirus (KSHV), vaccinia virus (VACV), murine gamma herpesvirus, herpes simplex virus type-1 (HSV-1), hepatitis B virus (HBV), and adenovirus 59, but also retroviruses such as human immunodeficiency virus (HIV), mouse leukaemia virus (MLV), simian immunodeficiency virus (SIV) 1, and West Nile virus (WNV) 60. In particular, the cGAS pathway is involved in many bacterial infections, such as those caused by *Listeria monocytogenes*, *Staphylococcus aureus*, *Burkholderia pseudomallei*, and *Pseudomonas aeruginosa* 61.

Most of the aforementioned DNA viruses and retroviruses either directly expose cells to their DNA or induce host cells to reverse transcribe viral RNA into DNA, which is recognized by cGAS and activates cGAMP, thereby activating STING in the ER to produce antiviral interferon-β (IFN-β) or other anti-inflammatory factors 1, increasing the number of invading pathogens eliminated. Some viral infections can elicit oxidative stress in host cells or induce IL-Iβ production, leading to the release of DNA from mitochondria into the cytoplasm, thereby enhancing the antiviral response and the production of Type 1 interferon 62-65. For bacteria, the Type 1 interferon production pathway can also be activated by mtDNA produced in response to bacterial LPS stimulation or by direct interaction of CDNs with STING 61, 66, 67. The role of STING activation in the production of IFN-β in resistance to DNA viruses is undeniable, but whether STING mediates host immune defence during RNA virus infection is still debated. RNA viruses are largely recognized by PRRs, including members of the Retinoic Acid Inducible Gene-I (RIG-I)-like receptor (RLR) or Toll-like receptor (TLR) families of proteins, and their downstream proteins Mitochondrial Antiviral Signalling (MAVS). These processes do not rely on STING to produce IFN-β 68. STING has been shown to play a protective role during RNA virus infection in certain cases and that its effect was IFN independent. Specifically, in fibroblasts, STING can prevent viral replication and inhibit viral protein expression, which results in an antiviral effect. This inhibitory effect is mediated through STING-induced translational inhibition, which does not require IFNβ production and does not depend on the cellular localization of STING. In mice resistant to HSV infection, STING is primarily associated with the effector function of T cells 69, 70. Autophagy is another mainstay of the STING-mediated antiviral infection response, which is IFN-β independent. During the transport of STING from the ER to the Golgi, STING-containing ERGIC acts as a membrane source for microtubule-associated protein 1 light chain 3 (LC3) lipidation via WIPI3 and ATG2, driving the formation of autophagosomes, which can then target pathogens and DNA that invade the organism and transport them to the lysosome for degradation 71 (Figure 1B). Interestingly, STING-mediated autophagy is probably the most conserved function of the STING pathway, predating the emergence of type I interferons.

Nonetheless, an immune escape strategy targeting cGAS or STING has evolved in many viruses to evade host immune system attack, either by directly degrading or binding key proteins in the pathway or indirectly interfering with signalling 59. For example, in HSV-1 infection, where USP21 mediates negative regulation of Type I interferon through the deubiquitination of STING 72, UL46 can both bind the N-terminus of STING 73 and inhibit the dimerization of TBK1 74 to deregulate the pathway. STING can be cleaved by the human dengue virus protease NS2B3 75 and the analogue DTMUV in duck tembusu virus 76, which disables the ability of cells to fight viruses. Through another mechanism, some viral shells protect viral DNA from leaking into the cytoplasm and being detected; this programme is evident in HBV 77. Understanding these viral escape strategies is critical for enhancing the efficacy of targeting STING in innate immunity and developing future antiviral immunotherapies.

## The cGAS-STING pathway in obesity

Adipose tissue is not only recognized as storage tissue for energy but is also considered an endocrine organ that can secrete leptin, adiponectin and other adipocytokines and regulate metabolic homeostasis. Changes in adipocyte characteristics, such as fat accumulation, increased expression of monocyte chemoattractant protein-1 (MCP-1), increased secretion of TNF-α and leptin, and decreased secretion of adiponectin, under conditions of obesity can lead to macrophage infiltration, which further aggravates disordered lipid metabolism, insulin resistance and inflammation spread 78.

**Figure 2:**
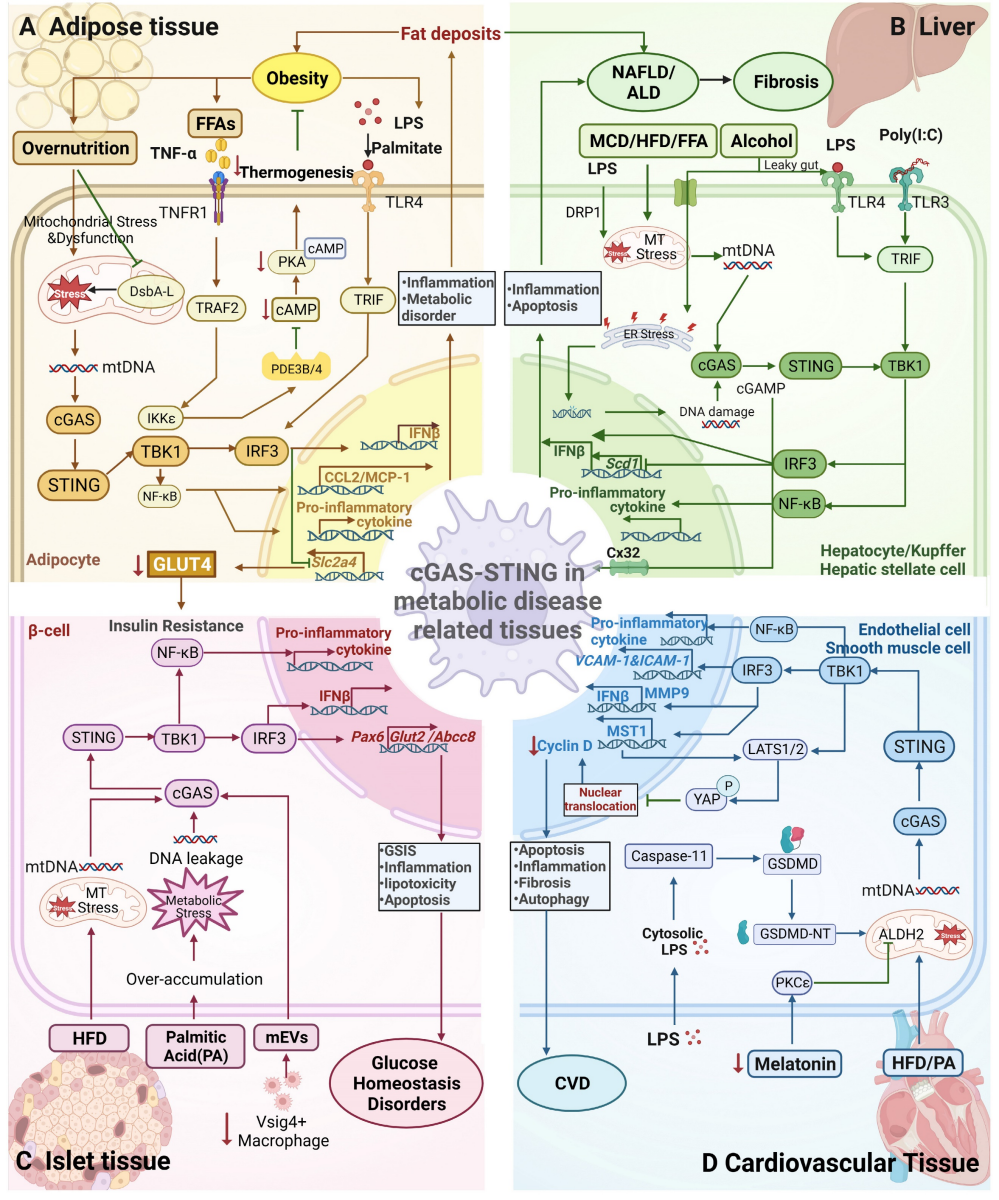
** Mechanism underlying STING signalling pathway activation in metabolic diseases. A) The cGAS-STING pathway in adipose tissue mediates obesity.** Overnutrition leads to mitochondrial stress (MT stress) and dysfunction, resulting in the release of mtDNA in adipocytes through DsbA-L inhibition. mtDNA is recognized by cGAS and activates the STING-TBK1-IRF3 pathway to produce Type I interferon or activate TBK1-NF-κB pathway, both of which leads to proinflammatory cytokine production. Similarly, LPS activates IRF3 by binding to TLR4 receptors. Activation of TNFR1 receptors by TNF-α and TLR4 receptors by palmitate synergistically increases CCL2/MCP-1 production, leading to macrophage infiltration and inflammation in the context of obesity. IRF3 inhibition of GLUT4 expression in adipocytes leads to insulin resistance. However, STING activation of TBK1/IKKε suppresses cAMP-PKA signalling by inhibiting PDE3B/4 expression, reducing thermogenesis, and ultimately attenuating obesity. **B) The cGAS-STING pathway in the liver mediates NAFLD/NASH/ALD.** MCD/HFD/free fatty acids (FFAs) and LPS cause MT stress and the release of mtDNA. The activation of mtDNA in hepatocytes/hepatic stellate cells is similar to that in adipocytes; it leads to increased inflammation, lipid accumulation and liver fibrosis through the production of proinflammatory cytokines. Transport of cGAMP from hepatocytes to immune cells via CX32 causes the spread of inflammatory effects. Alcohol-induced ER stress or activation of LPS/TLR4 signalling can activate the cGAS-STING-IRF3 pathway, resulting in apoptosis. However, IRF3 represses Scd1 transcription in response to poly (I:C)/TLR3/TRIF signalling, leading to the inhibition of adipogenesis. **C) The cGAS-STING pathway in islet tissue mediates glucose homeostasis.** Both HFD-induced mtDNA release and palmitic or PA-induced DNA leakage can activate the cGAS-STING pathway, which induces the production of Type I interferons and proinflammatory cytokines, causing inflammation, insulin resistance and lipotoxicity in islets. Additionally, a decrease in the number of Vsig4+ macrophages lead to the increased entry of mEVs into cells, thereby inducing the release of DNA, which activates the cGAS-STING-TBK1 pathway. However, IRF3 upregulates the expression of the transcription factor Pax6 to promote the expression of the target genes *Glut2* and *Abcc8*, resulting in GSIS and thus maintaining glucose homeostasis. **D) The cGAS-STING pathway in cardiovascular tissue mediates CVD.** HFD-/PA-induced increases in MT stress and reduced mitochondrial ALDH2 activity mediated by decreased melatonin levels lead to impaired mitochondrial function, causing the release of mtDNA. Activation of Caspase-11-induced GSDMD activation by LPS, leading to the release of mtDNA from damaged mitochondria. Subsequently, mtDNA activates the STING-IRF3 pathway, promoting macrophage infiltration and cardiac fibrosis. DNA fragmentation in SMCs drives DNA transfer to macrophages and promotes the expression of MMP-9, which contributes to extracellular matrix formation. Activated IRF3 enters the nucleus and induces the expression of MST1, which promotes YAP phosphorylation and suppresses the transcription of the cyclin D gene, leading to inhibition of endothelial cell angiogenesis. It also enhances the expression of ICAM-1 and VCAM-1, thereby increasing the inflammatory response and promoting SMC proliferation, thereby exerting a proatherogenic effect.

Accumulating evidence in recent years has emphasized the significance of the cGAS-STING signalling pathway in controlling sterile inflammation and energy homeostasis. Under conditions of obesity, the activation of the STING pathway leads to an inflammatory response, mainly in macrophages. Abe et al. 42 first reported that LPS or palmitic acid activated IRF3 and increased IFN-β expression in RAW264.7 (a mouse macrophage line) cell culture, and this outcome was mediated by TLR4. In HFD-fed obese mice, IRF3 knockout markedly reduced the number of infiltrating F4/80^+^ cells into white adipose tissue (WAT), reduced the number of M1 macrophages (CD11c^+^) and increased the number of M2 macrophages (CD301^+^) 43. Furthermore, increased expression of cGAS, STING and phosphorylated IRF3 has also been observed in macrophages 28. Through TLR4-TRIF and TNF-α/TNFR1 signalling, IRF3 in human monocytes, can contribute to monocyte infiltration and inflammation of adipose tissue 58 (Figure 2A). STING contributes to LPS-induced proinflammatory signalling in bone marrow-differentiated macrophages (BMDMs) 34. In addition, STING-IRF3 signalling, including type I IFN, contributes to the balance of biosynthesis and import of lipids by decreasing the lipid synthesis rate and increasing the amount of cholesterol and long-chain fatty acids imported into macrophages 79. Similarly, inhibition of TBK1 reduces macrophage infiltration and reduces the levels of mRNAs encoding key inflammatory genes in adipose tissue, which is accompanied by increased brown adipose tissue, lipid oxidation rates and energy expenditure 80, 81. These findings indicate crucial roles for IRF3 and TBK1 in regulating the immune response, adipocyte infiltration, and macrophage polarization in the context of obesity. The cGAS-STING pathway may be a mediator in the metabolic inflammatory circuit.

The cGAS-STING-mediated inflammatory response is found not only in immune cells under conditions of obesity but also in some metabolic cells. A p204-STING-mediated antiviral response involving the regulation of lipid metabolism in adipocytes was previously identified in adipose tissue. It promoted the proliferation of preadipocytes and inhibited leptin and lipocalin expression, and its expression was reduced in adipocytes 27. Increased expression of IRF3 has also observed in adipocytes from HFD-fed obese mice and in humans with obesity. In adipocytes, IRF3 activation by TLR3 or TLR4 was observed to be associated with the expression of LPS-induced inflammatory genes (e.g., Ccl6, Nos2). The reduced inguinal WAT (iWAT) mass and increased oxygen consumption in Irf3^-/-^ knockout mice may be due to white adipose browning to beige adipocytes, which increases thermogenesis 43. In addition to IRF3, TBK1, another component in the pathway, is involved in inflammatory metabolism in adipocytes. Increased expression and activity of TBK1 were observed in WAT and liver, and global TBK1 knockout reduced weight gain and the size of adipocytes in the WAT of HFD-fed mice 80. Adipocyte-specific TBK1 knockout (ATKO) reduced fat mass in HFD-fed obese mice, and ATKO increased energy expenditure by attenuating AMP-activated protein kinase (AMPK) inhibition but exacerbated adipose tissue inflammation by increasing the NF-κB level 82. This outcome may be due to the involvement of TBK1 in the negative regulation of AMPK and NF-κB expression in obese mice. These studies suggested that the specific effect of a high TBK1 level depends on different adipose tissues and the specific metabolic microenvironment in which it is located, which coincides with the different roles of inflammation in different stages and conditions of obesity 83.

In addition to its specific components, the whole canonical STING-IRF3 pathway is involved in adipose inflammation in the context of obesity. The role of STING in the proinflammatory signalling of adipocytes has been observed. STING significantly enhanced the phosphorylation of Jun N-terminal kinase (JNK) p46 and NF-κB p65 in response to LPS stimulation in differentiated 3T3-L adipocytes and primary hepatocytes 34. Furthermore, the specific deficiency of disulfide bond A oxidoreductase-like protein (DsbA-L) in adipose tissue and adipocytes in the obesity context led to mitochondrial dysfunction. This dysfunction resulted in the release of mitochondrial DNA (mtDNA), which activated the cGAS-STING pathway, subsequently triggering phosphodiesterase 3B/4 (PDE3B/4). This was followed by a further reduction in cAMP levels and protein kinase A (PKA) signalling, which contributed to the obesity and inflammation caused by a high-fat diet 28, 84 (Fig. 2A). In addition, ICAM-1 (intracellular adhesion molecule 1) expression in endothelial cells was also regulated by STING-IRF3 signalling, which promoted macrophage infiltration and HFD-induced endothelial inflammation in adipose tissue 28. In summary, overnutrition, cytokines and cytosolic DNA can activate broad-spectrum cGAS-STING signalling in different cell types of adipose tissue under conditions of obesity, thereby contributing to inflammation and influencing energy metabolism.

## The cGAS-STING Pathway and Nonalcoholic Fatty Liver Disease and Alcoholic Liver Disease

NAFLD, a common complication of obesity, encompasses a range of liver conditions, from fatty liver (steatosis) to liver inflammation (nonalcoholic steatohepatitis, NASH). NASH can progress to severe outcomes such as cirrhosis, hepatocellular carcinoma, and liver failure, increasing mortality in obese patients 85. The vital role of the cGAS-STING signalling pathway in NAFLD/NASH has been clearly demonstrated. However, the expression of components and the specific role of the pathway in the liver are unclear. Some evidence suggests that STING is expressed in nonparenchymal cells 54, 86, while other findings show that STING is expressed in hepatocytes and involved in hepatocyte injury and apoptosis 87, 88.

Contradictory effects of the role played by IRF3 on NAFLD were reported in different studies. Previous studies showed that IRF3 in the cytoplasm can interact with the structural kinase domain of IKKβ and inhibit downstream NF-κB activation. Thus, reduced levels of IRF3 in hepatocytes from fatty livers may protect mice from HFD-induced hepatic steatosis, inflammation and macrophage infiltration 89. Another study reported that IRF3 inhibited TLR/TRIF-mediated adipogenesis in hepatocytes at the transcriptional level by directly binding to the Scd1 promoter (stearoyl coenzyme A (CoA) desaturase 1, the gene encoding the rate-limiting enzyme for adipogenesis) 57 (Figure 2B). In contrast, the STING-IRF3 pathway showed greater effects on lipid accumulation, inflammation and apoptosis in hepatocytes 90. The aforementioned study demonstrated significantly elevated levels of STING and IRF3 in the livers of HFD-fed obese mice and L-O2 cells (human hepatocytes), accompanied by high levels of p-p65/p65, inflammatory cytokines and markers of apoptosis in the liver. Knocking down either STING or IRF3 by siRNA significantly reduced in the inflammation and apoptosis rates, increased the levels of stored glycogen, and attenuated lipid accumulation. Different upstream signalling pathways may contribute to different roles of IRF3 in hepatocytes, in which, for example, TRIF-IRF3 inhibits lipogenesis while STING-IRF3 aggravates inflammation.

Many studies suggested that STING-IRF3 can aggravate NAFLD/NASH by increasing liver fat accumulation and the proinflammatory response. Elevated TBK1 phosphorylation accompanied by increased proinflammatory signalling was observed in the livers of HFD-fed mice 34. Inhibiting TBK1 by administering amlexanox or knocking out the TBK1-encoding gene significantly alleviated hepatic steatosis by decreasing fat accumulation, lipogenic gene expression, proinflammatory cytokine levels and macrophage infiltration in the livers of HFD-fed mice or ob/ob mice 80,81. Cho et al. 91 showed that the cGAS-STING-TBK1 pathway was activated and mediated p62 phosphorylation and protein inclusions (a critical marker of NASH) in saturated fatty acid (SFA)-treated homoplastic hepatocytes and HFD-fed obese mice. In a mouse NASH model, STING and TLR9 synergistically promoted hepatocyte mtDNA-induced inflammatory responses via the action of Kupffer cells (KCs), in which STING may regulate glucose levels via the IRF3 pathway independent of its regulatory effect on body weight 92.

Notably, recent research confirmed that the cGAS-STING pathway in macrophages may play a prominent role in NAFLD. First, activating STING by DMXAA increased IFN-β production in BMDMs and 3T3-L1 adipocytes but not in primary hepatocytes in mice 34. Second, STING-TBK1-IRF3 was activated in the livers of HFD-fed mice and NAFLD patients, and only STING in BMDMs exacerbated the severity of HFD-induced NAFLD. An *in vitro* study confirmed that hepatocyte fat was deposited in a BMDM STING-dependent manner but not in hepatocytes 54. STING activation in Kupffer cells might be related to mtDNA release. In mice with LPS-induced septic liver injury, dynamin-related protein 1 (DRP1) induced mitochondrial fission and excessive oxidative stress in Kupffer cells, accelerating mtDNA leakage, which is required for STING activation 86. However, during NAFLD/NASH, the role of STING in different types of macrophages remains to be studied. A population-based trial in 2020 confirmed that STING expression in mononuclear cell-derived macrophages (CCR2+, S100A9+) and liver-resident macrophages (KCs) was significantly increased during the progression of NAFLD, particularly NASH, whereas STING expression was increased only in KCs during the progression of NASH to cirrhotic liver cancer 93.

STING also promotes the progression of liver fibrosis. STING knockout decreased MCD diet-induced expression of fibrosis markers (αSMA, TGF-β1 and Collagen 1A1) and inhibited liver fibrosis induced by chronic inflammation in mice 54. STING activation increased TGF-1-induced fibrosis in human hepatic stellate cells (HSCs), the main cells driving fibrosis. Furthermore, activated STING in wild-type BMDMs in culture or in HSCs treated with BMDM-conditioned medium caused TGF-β1-induced fibrosis in a STING-dependent manner. It has been hypothesized that STING in macrophages triggers liver fibrosis through paracrine signalling to activate HSCs 54. Whether STING in HSCs directly promotes fibrosis is still unclear. In addition, some findings demonstrated that STING-IRF3 linked hepatocyte death caused by mitochondrial stress to liver fibrosis 88. This connection partly explains why liver fibrosis is not only driven by inflammation but is also affected by secondary liver injury caused by hepatocyte death 88.

Alcohol-related liver disease (ALD), which can range in severity from mild steatosis to liver fibrosis, cirrhosis, and even liver cancer, is caused by the interaction of alcohol-induced hepatotoxicity and oxidative stress. There is no effective clinical treatment for the condition 94. IRF3 responds to ER stress in hepatocytes during the early stages of the AID process. Then, activated IRF3 is transported to mitochondria along with the apoptotic protein Bax to initiate STING-mediated apoptosis in hepatocytes 95. Alcohol induces the release of mitochondrial DNA in hepatocytes to activate cGAMP. Transport of cGAMP by connexin 32 (Cx32) in the intercellular space enables the transfer of injury- and inflammation-promoting signals from hepatic parenchymal cells to hepatic nonparenchymal cells 96 (Figure 2B). The activation of STING resulted in a robust secondary inflammatory response in alcoholic fatty liver. In conclusion, cGAS-STING is necessary for nutrient stress-induced NAFLD/NASH and even hepatocellular carcinoma 97. The cGAS-STING effect is mainly mediated through macrophages and hepatocytes, and therefore cGAS-STING pathway shows great potential to be a therapeutic target.

## The cGAS-STING pathway and glucose homeostasis

Impaired glucose homeostasis is a common obesity-related metabolic disorder. The decrease in insulin secretion from pancreatic β-cells and acquired insulin resistance in adipose tissue, liver and muscle are the main causes of hyperglycaemia. Proinflammatory factors expressed under obesity conditions has been identified as a major cause of glucose metabolism disorders, in which innate immunity signalling plays an essential role. The cGAS-STING pathway participates in inflammation and interacts with the insulin signalling pathway, further regulating glucose homeostasis.

In an early study, IRF3 exerted protective effects on mice with diet-induced hepatic insulin resistance 89. The interaction of IRF3 with IKKβ inhibited proinflammatory IKKβ/NF-κB signalling in the liver, mitigating insulin resistance. IRF3 knockout reduced the phosphorylation of residue Tyr608 in insulin receptor substrate 1 (IRS1) and the phosphorylation of protein kinase B (AKT) in the livers of HFD-fed mice, further aggravating HOMA-IR and insulin resistance 89. In contrast, in cultured 3T3-L1 adipocytes, IRF3 was needed for LPS (TLR4 ligand)- or polyinosinic-polycytidylic acid (poly (I: C)) (TLR3 ligand)-induced insulin resistance 43. In this study, IRF3 knockout improved glucose homeostasis in HFD-fed mice by increasing glucose uptake, the expression of *Slc2a4* (encoded by glucose transporter 4, GLUT4), and insulin-stimulated phosphorylation of p70S6K (T389) and AKT (S473) in iWAT, which directly regulated the insulin signalling pathway 43 (Figure 2C). The role of IRF3 in insulin resistance varies by tissue, and further study with tissue-specific IRF3 knocked out is needed. IRF3 is also involved in islet function and insulin secretion. In INS-1 cells (a rat insulinoma cell line), cGAS-STING promoted the apoptosis of INS-1 through the action of apoptotic proteins BAX cysteine-3 and PARP, demonstrating the role of STING-IRF3 in islet β-cell lipotoxicity 98. More evidence should be obtained to confirm the role of STING-IRF3 in islet function.

TBK1 directly phosphorylated insulin receptor (IR) on serine (Ser) 994 in the liver of obese Zucker rats or HFD-fed mice, inhibiting insulin signalling pathway activation and promoting insulin resistance 80, 99. Inhibiting TBK1 by amlexanox increased the insulin-stimulated phosphorylation rate of AKT, increased insulin sensitivity and decreased glucose tolerance in HFD-fed mice and ob/ob mice 81 as well as in patients with T2DM 100. In this procedure, the liver was targeted to improve insulin sensitivity. Interestingly, TBK1 was shown to be related to insulin signalling in the brains of obese mice 101. Recruitment of TBK1 into lipid rafts (LRs) and postsynaptic density (PSD) fractions was observed to be accompanied by decreased insulin receptor and Akt levels in the hippocampus of ob/ob obese mice. An *in vitro* study showed that recruitment of TBK1 correlated with the inhibition of insulin signalling in palmitic acid-treated cortical mouse neurons 101. In contrast, ATKO aggravated insulin resistance in adipose tissue by increasing macrophage infiltration, and these effects involved the negative regulation of NF-κB 82. These results suggested that the effects of TBK1 on insulin resistance may be tissue specific, which needs to be confirmed in future studies.

STING also acts as a promotor in HFD-induced insulin resistance. STING disruption attenuated systemic insulin resistance and glucose intolerance in HFD-fed mice 29, 54, and myeloid cell-specific STING disruption was sufficient to alleviate insulin resistance 54. Moreover, the activation of the cGAS-STING pathway by increasing mtDNA release (DsbA-L deficiency) aggravated systemic insulin resistance in HFD-fed mice 28. Global STING deficiency alleviated HFD-induced insulin resistance and glucose intolerance in peripheral tissues such as adipose and liver tissues. Nonetheless, defective STING in β-cells reduced the expression of the key transcription factor Pax6 in β-cells, disrupting its binding to target gene promoters in the nucleus and leading to glucose-stimulated insulin secretion (GSIS) 102 (Figure 2C). Nevertheless, the significant insulin resistance associated with whole-body STING knockout was able to counteract the negative effects of GSIS. Future research should focus on regulating STING expression to maintain glucose metabolism balance of insulin in target tissues and β-cells.

Impaired intestinal barriers in people with obesity have been linked to β-cell dysfunction of islet tissue. Microbial DNA-containing extracellular vesicles (mEVs) can pass through the damaged barrier and infiltrate β-cells, causing inflammation and dysfunction via the cGAS-STING pathway. Vsig4+ macrophages in islet tissue protects β-cells from mEV infiltration, but obesity reduces their population. Thus, clearing CD11c+ macrophages and restoring Vsig4+ macrophages may reduce microbial DNA-induced β-cell inflammation and abnormalities 103. The cGAS-STING pathway is also involved in the senescence of β-cells caused by metabolic stress. The pathway activates downstream factors such as TBK1 and Type I interferons and induces senescence-associated secretion profile (SASP) secretion, leading to β-cell cycle arrest and the inability of cells to compensate for damage 104. In brief, components of the cGAS-STING pathway interact with inflammatory and insulin signalling pathways in a tissue-specific manner to regulate insulin response and glucose homeostasis.

## The cGAS-STING pathway and cardiovascular diseases (CVDs)

CVDs, including heart failure, myocardial infarction (MI), coronary artery disease, and aortic aneurysm and dissections (AAD), are the leading causes of death globally 105, and they are linked to metabolic dysregulation and inflammation caused by overnutrition and obesity 106. Alternatively, atherosclerosis, myocardial hypertrophy, and hypertension drive CVDs by causing inflammation and repair responses in endothelial cells after mitochondrial damage and an increase in ER stress, unfolded proteins, and macrophage infiltration, leading to fibrosis in severe cases 29, 107. The cGAS-STING pathway promotes atherosclerosis and cardiac injury and inhibits angiogenesis, causing inflammatory reactions in endothelial cells and macrophages.

IRF3 promotes the development of atherosclerosis in humans and mice 56. Upregulation of IRF3 expression has been observed in the endothelial cell layer of atherosclerotic arteries and macrophages in atheromatous plaques from patients with coronary heart disease and hyperlipidaemic ApoE^-/-^ mice (mice with HFD-induced atherosclerosis). IRF3 knockout significantly decreased the development of atherosclerosis in hyperlipidaemic ApoE^-/-^ mice, which reduced necrotic core size, macrophage infiltration, lipids, and inflammation. Moreover, VCAM-1 (vascular cell adhesion molecule-1) and ICAM-1 expression was suppressed in endothelial cells, which attenuated macrophage infiltration. Furthermore, IRF3 depletion in endothelial cells was more important for reducing atherosclerosis than its depletion in bone marrow-derived haematopoietic cells 56 (Figure 2D). Mitochondrial damage and leakage of mtDNA trigger cGAS-STING signalling in primary human aortic endothelial cells (HAECs), leading to ICAM-1 expression and monocyte-endothelial cell adhesion, further promoting atherosclerosis 29, 56. Bacteria-derived LPS activated Caspase-11, which cleaved Gasdermin D (GSDMD), causing the insertion of the N-terminal domain of GSDMD (GSDMD-NT) into mitochondria, resulting in mitochondrial damage and the release of mtDNA to activate the cGAS-STING signalling pathway. Inhibition of endothelial cell angiogenesis can be stimulated by the direct binding of phosphorylated IRF3 to the mammalian Ste20-like kinase 1 (MST1) promoter, which leads to Yes-associated protein (YAP) inactivation and suppresses transcription of cyclin D genes. It can also stimulate large-scale tumour suppressor kinase (LATS)-mediated YAP degradation due to an increased cGAMP concentration 30, 67. The cGAS-STING pathway-mediated proinflammatory response in macrophages has also been identified as a potential target in patients with atherosclerosis 56, 108. Both cGAMP and mtDNA activated TBK1 and NF-κB in the macrophages of the aorta of atherosclerotic mice, but cGAS was not the only DNA sensor involved. Despite these findings, the relationship between DNA damage and atherosclerosis remains unclear, and the mechanisms leading to DNA damage are not yet fully understood.

AAD is a fatal disorder characterized by increasing aortic smooth muscle cell (SMC) loss and extracellular matrix degradation. STING has been shown to be highly expressed in SMCs and macrophages in human AAD tissues. In the STING-deficient (STING^gt/gt^) mouse model of sporadic aortic aneurysm, a significant reduction in challenge-induced aortic enlargement, entrapment and rupture was observed in both thoracic and abdominal aortic regions, indicating that STING is involved in aortic degeneration and the formation of AAD. Furthermore, STING promotes degenerative aortic changes through SMC apoptosis and necrosis, involving STING-induced Type I interferon and tumour necrosis factor receptor signalling. Injured SMCs release DNA into macrophages to activate STING-mediated IRF3-MMP-9 signalling to degrade the extracellular matrix 109 (Figure 2D). The reason for DNA leakage into the cytoplasm of injured SMCs and how this DNA is transmitted both inside SMCs themselves and to macrophages need to be further studied.

In addition, TBK1-IRF3 aggravates the fructose-induced injury of cardiac muscle cells 110. NLRP4, a nod-like receptor protein, can reduce the expression of TBK1/IRF3 and curb inflammation and hypertrophy. STING knockdown alleviated aortic banding-induced cardiac hypertrophy in mice and inhibited the release of inflammatory factors and fibrosis formation in cardiomyocytes. ER stress was shown to participate in STING-regulated pathological cardiac hypertrophy 107. During myocardial infarction, activation of STING in macrophages is responsible for inflammatory infiltrates, fibrous deposits and apoptosis in cardiomyocytes and cardiac fibroblasts 31. For HFD-induced cardiac remodelling and contractile abnormalities, excessive activation of cGAS-STING signalling mediated an increase in inflammation and defective mitochondrial autophagy 111. In Alzheimer's disease (AD)-induced cardiac abnormalities, melatonin treatment was shown to ameliorate myocardial remodelling and contractile abnormalities through the restoration of cGAS-STING-TBK1 signalling pathway-mediated disruption of mitochondrial integrity in mice via the mitochondrial aldehyde dehydrogenase ALDH2 112. Interestingly, the results of this study suggested that excessive mtDNA leakage abrogated cGAS-STING signalling, contrary to conventional beliefs. Effective STING activation may depend on mitochondrial integrity and the intracellular cGAS level, and excessive mtDNA may deplete intracellular cGAS and inhibit STING activity. Further evidence is needed to confirm this possibility. In summary, activation of the cGAS-STING pathway is involved in cardiovascular disease through a variety of cells, mediating inflammation and macrophage infiltration and participating in angiogenesis. Future studies can be directed to investigate the proportional contributions of the pathway to endothelium and immune cell activity in the contexts of CVDs.

## Cross-talk with other signalling pathways in metabolic disease

### TLR4-NF-κB

**Figure 3:**
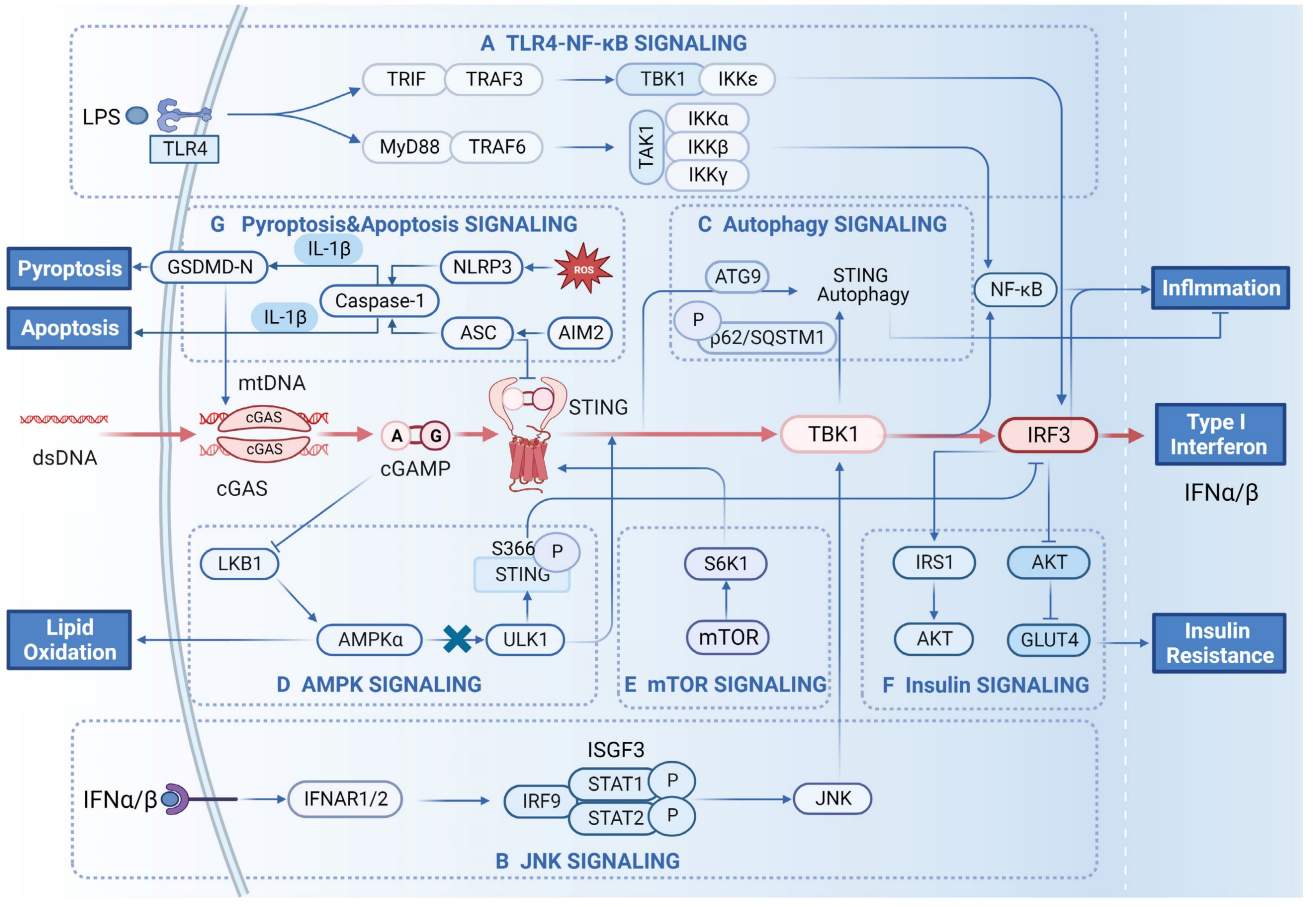
** The signalling network between cGAS-STING signalling and other signalling pathways. A) Crosstalk with TLR4-NF-κB signalling.** Recognition of bacterial LPS by TLR4 receptors activates downstream signalling pathways. Activated TLR4 binds MyD88 and activates downstream TRAF6, leading to TBK1/IKK-mediated activation of NF-κB. TLR4 also activates the TRIF-dependent pathway by binding TRIF, which activates TBK1 and IKKε, ultimately leading to IRF3 activation and IFN-β production. **B) Crosstalk with JNK signalling.** The type I interferon IFN-α/β produced by the cells can in turn target the IFNAR receptor, which activates STAT1 and STAT2 and forms the transcription factor complex IFN-stimulated gene factor 3 (ISGF3). ISGF3 can activate the JNK/TBK1/IRF3 signalling cascade. **C) Crosstalk with autophagy signalling.** Activated STING can be degraded in lysosomes in response to the autophagy protein ATG9. STING can also be degraded by phosphorylation of p62/SQSTM1. All of these modalities can inhibit the inflammatory response resulting from the sustained activation of STING.** D) Crosstalk with AMPK signalling.** The cGAMP generated by cGAS inhibits the activation of LKB1/AMPKα, resulting in the dissociation of ULK1 from AMPK. Subsequently, ULK1 phosphorylates the S366 site of STING, which inhibits IRF3 and reduces the production of IFN-β. **E) Crosstalk with mTOR signalling.** The downstream effector of mTOR, S6K1, can form the S6K1-STING-TBK1 complex and promote the activation of IRF3. **F) Crosstalk with insulin signalling.** Activated IRF3 inhibits the expression of AKT and the downstream signalling molecule GLUT4, which causes glucose accumulation in adipose tissue. In contrast, it can also stimulate glucose uptake and utilization in adipose tissue through the AKT/IRS1 pathway. **G) Crosstalk with cell death signalling pathways.** MT stress induces the release of ROS, which trigger the NLRP3/caspase-1/GSDMD-mediated release of mtDNA into macrophages, thereby activating the cGAS-STING pathway. This can produce proinflammatory cytokines, leading to hepatocyte pyroptosis. Bacterial DNA activates AIM2/Caspase-1-mediated apoptosis, resulting in elevated expression of ASC proteins. After activation, ASC interacts with STING to block the activation of TBK1 and IRF3, thereby inhibiting the production of IFN-β.

The proinflammatory role of TLR4-NF-κB signalling in the context of obesity has been well established 113. With the discovery that the TIR domain-containing adaptor inducing interferon-β (TRIF), which is protein linking TLR3 with TLR4, has been shown to directly interact with STING and promote its dimerization 114, the link between Toll-like receptors and the STING pathway is slowly emerging. TLR4 has been observed upstream of IRF3 and controls the activation of IRF3, which is related to the expression of LPS-induced inflammatory genes in adipocytes or macrophages. TLR4-TRIF-IRF3 signalling is responsible for palmitate/TNF-α-stimulated MCP-1 (chemokine (C-C motif) ligand 1, CCL2), a key regulator of monocyte infiltration into adipose tissue, expression in human monocytes, resulting in adipose tissue inflammation 58. Moreover, TRIF has also been observed upstream of IRF3, promoting IRF3 binding to the SCD1 promoter, and resulting in reduced TG levels in HepG2 cells 57. The activation of IRF3 by TLR4 may be dependent on TBK1, which is similar to the activation of this pathway in the innate immune response 40. In 2015, LPS was shown to trigger IFN-I production and activate interferon receptor (IFNAR) signalling to induce cGAS expression in BMDMs via a TRIF-dependent pathway, and the activation of cGAS by IFN-I constituted a positive loop in the cGAS-STING pathway 115. Subsequently, it was further demonstrated that LPS upregulated cGAS expression via the myeloid differentiation factor 88 (MyD88)-independent TLR4-STING signalling pathway 116 (Figure 3A). NF-κB participates in complex interactions with the STING-TBK1-IRF3 pathway. In the innate immune response, STING synchronously activates IRF3 and the kinase IKK, which phosphorylates the IκB family and leads to the activation of NF-κB 2. Under conditions of obesity, NF-κB activation by STING has been observed in adipocytes 28, 34 and hepatocytes/liver tissue 54, 90, resulting in significant proinflammatory effects. IRF3 has also been observed to interact with the kinase domain of IKKβ in the cytoplasm and inhibit IKKβ Ser181 phosphorylation and activation in hepatocytes, leading to the constraint of NF-κB signalling and alleviating HFD-induced hepatic inflammation 89. TBK1 has been reported to inhibit NF-κB by phosphorylating NF-κB-inducing kinase (NIK) and inducing its degradation to attenuate NF-κB activation. ATKO promoted HFD-induced inflammation and insulin resistance in adipose tissue by stimulating NF-κB activity 82. This process is also critical for the inhibition of NF-κB by activating AMPK-Unc-51-like protein kinase 1 (ULK1)-TBK1 under caloric restriction conditions 82. Based on previous studies, the interaction of TLR4-NF-κB with components in the STING-IRF3 pathway results in different outcomes in obesity-related metabolic disorders, and the mechanisms underlying these outcomes have not been fully elucidated.

### JNKs

JNKs, constituting one subfamily of the mitogen-activated protein kinase (MAPK) group of serine/threonine protein kinases, are activated by inflammatory cytokines and free fatty acids and promote inflammation and insulin resistance in the context of obesity 117. Activation of JNK is necessary for the phosphorylation of IRF3 by TBK1 in antigen-presenting cells during adenovirus infection. Moreover, activated IRF3 and Type I interferon are needed for the activity of JNK 118 (Figure 3B). These results reveal the important interaction of IRF3 with JNK in immune responses. Activated STING has also been reported to enhance LPS-induced phosphorylation of JNK in adipocytes 34. Global or myeloid cell-specific STING disruption resulted in significantly decreased JNK phosphorylation in the livers of HFD-fed NAFLD mice 54. Further studies can be focused on the interactive pattern of JNK (or other kinases in the MAPK group) with cGAS-STING in patients with obesity.

### Autophagy and AMPK

The interaction of STING with autophagy proteins regulates the activity of STING-IRF3 signalling during the innate immune response, especially after the appropriate deactivation of the STING signalling function. The autophagy proteins LC3 and autophagy-related protein 9 (ATG9) colocalize with STING to TBK1 after dsDNA stimulation. The suppression of ATG9 resulted in the persistent activation of STING-TBK1 by dsDNA, leading to an aberrant innate immune response 119 (Figure 3C). The autophagy kinase ULK1 has been observed to phosphorylate STING on Ser366 and suppress STING-IRF3 but not NF-κB. In this process, ULK1 is activated via its disassociation from AMPK, which is activated by cyclic dinucleotides 120. Interestingly, phosphorylation of STING on Ser366 is responsible not only for STING-IRF3 activation by TBK1 but also for STING degradation by ULK1 after autophagy has been initiated 120 (Figure 3D). Competitive phosphorylation may lead to the progressive effects of TBK1 and ULK1, and the mechanism remains unclear. The interaction of TBK1 with AMPK/ULK1 affects the inflammation status and energy homeostasis in adipose tissue. Under conditions of obesity, TBK1 directly phosphorylated AMPKα1 on Ser459 and Ser476 and inhibited AMPK activity to repress respiration and increase energy storage 82. On the other hand, activated AMPK (via activator or caloric restriction) resulted in the phosphorylation of TBK1 at Ser172 via the AMPK downstream target ULK1, which mediated the negative impact of AMPK on NF-κB activation 82. In addition, IFR3 is also regulated by AMPK signalling. Adiponectin has been reported to induce the expression of IRF3 via AMPK, and impaired adiponectin-AMPK resulted in reduced IRF3 expression in programmed male offspring from rat dams fed a low-protein diet 121. Metformin, a well-known AMPK activator, increased the expression of IRF3 in WAT, which was suppressed in* db/db* mice 122.

### mTOR

Mammalian target of rapamycin (mTOR), belonging to the phosphoinositide 3-kinase (PI3K)-related kinase family, is an important regulator of metabolism, energy homeostasis and nutrition 123. mTOR complex 1 (mTORC1) and 2 (mTORC2) are two major components of the mTOR signalling pathway that show different sensitivities to rapamycin. mTORC1 is a sensor of amino acid levels, is activated by overnutrition and contributes to obesity and insulin resistance 124. S6 kinase 1 (S6K1), the downstream effector of mTORC1, interacts with STING and form a tripartite complex with STING and TBK1, which is required for DNA virus-induced IRF3 activation in immune cells 125 (Figure 3E). The interactions of TBK1 with mTORC1 resulted in different responses in previous studies. Bodur et al. 126 reported that TBK1 directly activated mTORC1 through site-specific phosphorylation (on S2159) in response to EGF-receptor or TLR3/4 activation in cultured cells. In macrophages, TBK1 activated mTORC1 signalling and promoted IRF3 nuclear translocation and IFN-β production after mTORC1 S2159 phosphorylation, thereby regulating inflammation 126. In contrast, Kim et al. 127 showed that TBK1 inhibited the activity of mTORC1 by blocking its phosphorylation at p70S6K in prostate cancer cells. A recent study elucidated that the interaction of mTORC1 with STING-TBK1-IRF3 regulated inflammation and metabolism in models with a background of three prime repair exonuclease 1 (Trex1) knocked out 32. Trex1 knockout activated TBK1, which inhibited mTORC1 activity, leading to reduced fat in storage and increased energy expenditure. In the models with a Trex1 knockout background, STING-TBK1-IRF3 was responsible for inflammation, while inhibition of mTORC1 by STING-TBK1 led to metabolic defects 32. Compared to the activation of STING by other signalling molecules to aggravate diet-induced obesity, Trex1 knockout-induced STING activation seems to result specifically in the inhibition of mTORC1, alleviating obesity; the mechanism underlying this obesity-reducing effect should be elucidated in the future.

### Insulin signalling

Canonical insulin signalling involves insulin receptor, IRS1, PI3K, AKT and GLUT4. The phosphorylation-triggered signalling cascades mediated by these components control GLUT4 translocation, leading to intracellular glucose transport. In the context of obesity, the interaction of insulin signalling pathways with other pathways causes dysfunction in insulin signalling and increased insulin resistance 128. Some evidence has shown that STING-IRF3 may interact with components of the insulin signalling pathway and regulate insulin resistance under conditions of obesity. IRF3 knockout reduced the phosphorylation of AKT and IRS1 Tyr608 in the livers of HFD-fed mice, leading to impaired insulin signalling 89 (Figure 3F). In contrast, another study showed that IRF3 knockout increased the expression of GLUT4 and promoted insulin-stimulated phosphorylation of AKT (S473) in iWAT, sustaining glucose homeostasis in HFD-fed mice 43. TBK1 directly phosphorylated insulin receptor (IR) on Ser994 in the liver of obese Zucker rats or HFD-fed mice, promoting insulin resistance 80,99 while inhibiting TBK1-enhanced insulin-stimulated phosphorylation of AKT 81. TBK1 has also been associated with decreased insulin receptor and AKT expression in the hippocampus of ob/ob mice 101. More studies are needed to elucidate the mechanisms underlying STING-IRF3 interactions with insulin signalling pathway components.

### Cell pyroptosis and apoptosis

Cell pyroptosis and apoptosis are two important forms of cell death. Cell pyroptosis is a proinflammatory form of programmed cell death. Moderate focal pyroptosis can lead to the recruitment of inflammatory cells that infiltrate the tumour microenvironment to suppress tumours or that reach the site of infection to clear pathogens, whereas excessive pyroptosis may lead to sustained inflammation and metabolic disturbance. Apoptosis is another form of the programmed cell death. Recent research has shown a relationship between the STING protein and cell death. In 2017, Gaidt et al. 129 revealed for the first time that in human bone marrow cells, DNA-mediated inflammasome activation was regulated by the cGAS-STING axis, triggering lysosomal cell death and further leading to NOD-, LRR- and pyrin domain-containing protein 3 (NLRP3) inflammasome-mediated cell pyroptosis. Nevertheless, the means of instigating cell pyroptosis induced by the STING pathway can vary in different pathological states and cell types. In neutrophils from septic mice, overexpression of N-acetyltransferase 10 (NAT10) upregulated the expression of ULK1, inhibited the activation of STING-IRF3 signalling and attenuated NLRP3 inflammatory vesicle-mediated apoptosis 130. Furthermore, cardiomyocyte and microglial pyroptosis were explained as cGAS-STING-NLRP3 axis-dependent cell pyroptosis (CVST) 131. DNA-activated AIM2 inflammasome-mediated cell apoptosis and STING activation in the same cells led to reduced STING-mediated IFN expression. This finding connects caspase-1-dependent cell pyroptosis with STING signalling, probably because pyroptotic cells have less viability and fewer intracellular inflammatory response pathways 132 (Figure 3G). In hepatocytes, NLRP3/caspase-1/GSDMD-mediated mitochondrion-mediated pyroptosis triggered by XBP1 deficiency, leading to the release of mtDNA into macrophages to activate cGAS-STING. The inflammatory response of macrophages may further exacerbate hepatocyte injury, forming positive feedback regulation 133.

## Small-molecule inhibitors targeting the cGAS-STING pathway for disease intervention

Reliable evidence highlights the considerable role of the cGAS-STING signalling pathway in exacerbating metabolic disorders, even though it is essential to antiviral and antitumour defences. There is an urgent need to develop inhibitors of pathway components and make them powerful tools for attenuating metabolic diseases. To date, some researchers have developed inhibitors of cGAS (Table 1), STING (Table 2) or TBK1 (Table 3). Various inhibitors regulate cell physiological effects based on different mechanisms, and some of these inhibitors have shown success in inhibiting inflammation, attenuating metabolic diseases and inhibiting tumorigenesis in animals. However, the effect of inhibitors has not been verified in the human body, and the effective dose and toxicology are not completely clear. In the future, a large number of studies, especially clinical studies, are needed to find disease treatments targeting the STING signalling pathway. This summary explains the dose, mechanism and physiological effects of inhibitors. Specifically, we summarize the small-molecule inhibitors that have been discovered thus far according to the different checkpoints in the STING pathway.

## Conclusions and prospects

Accumulating evidence suggests that crosstalk between metabolic signalling and immune responses plays a large role in maintaining energy homeostasis. The capacity of the cGAS-STING pathway to maintain activity and stability during antiviral and anticancer activities illustrates its dual effectiveness. Although the transient and rapid activation of this pathway reveals its high antitumour and antiviral efficacy, the key proteins within this pathway are highly prone to degradation or epigenetic alterations. These changes ultimately culminate in the attenuation of their protective attributes when challenged due the complex array of defence mechanisms it can trigger. Therefore, it is crucial to acknowledge that achieving precise control over the temporal dynamics and magnitude of cGAS-STING signalling pathway activation remains a challenging endeavour. Sustained activation of the pathway can potentially aggravate inflammation-driven metabolic disorders and tumorigenesis. The ultimate outcome may be contingent upon the specific cellular location and the type of cell in which STING is activated, such as tumour cells or nontumour cells, including adipocytes, metabolic cells and immune cells. Hence, it is paramount to emphasize the tissue-specific and cell-specific attributes of STING activation for a meticulous dissection of the distinctive pathological traits characterizing various diseases, which may enable the development of tailored and refined interventions. Prominent research findings have substantiated the clear exacerbating impact of cGAS-STING signalling on metabolic disorders. Activation of cGAS-STING has been observed not only within metabolic cells such as adipocytes, hepatocytes, and endothelial cells but also in macrophages and neurons. The activation of this pathway leads to macrophage infiltration, proinflammation and fat accumulation in adipose tissue, liver, vascular endothelium and the hypothalamus, resulting in the aggravation of obesity, NAFLD, insulin resistance and atherosclerosis. Overnutrition, mtDNA, cGAMP or LPS can stimulate cGAS-STING and activate downstream effectors, such as proinflammatory cytokines, monocyte infiltration regulators and metabolism-related proteins. The components of the cGAS-STING pathway can interact with TLR4-NF-κB, JNK, autophagy, AMPK, mTOR, and insulin signalling pathways and thus regulate cellular inflammation and metabolism. Targeting dysfunction STING and blocking its signalling, therefore, offers new possibilities for the treatment of chronic inflammatory metabolic diseases. In summary, given the high expression and prominent involvement of STING signalling molecules within cells associated with metabolic diseases, coupled with the multitude of disease-related molecular targets both upstream and downstream of STING, it is imperative to delve into the molecular mechanisms governing the role of STING in metabolic diseases. Furthermore, when contrasted with the direct manipulation of downstream interferon signalling molecules, the direct regulation of components within the cGAS-STING signalling pathway might be a less intricate and risky approach. However, it is important to acknowledge that there are still certain limitations in our understanding of the role of the STING signalling pathway in metabolic diseases. The regulation of the STING signalling pathway is intricately influenced by many factors, including the specific tissue where it is activated and the nutritional status of the cell. Pinpointing and identifying the sources of STING activation and developing drugs that can precisely target and inhibit this activation are significant challenges. Moreover, the extent of the risk associated with inhibitors targeting the STING pathway remains uncertain when compared to inhibitors of other interconnected upstream and downstream targets, and the challenges related to targeting this pathway in humans and the potential side effects remain unverified. Therefore, in addition to summarizing the small-molecule inhibitors, the article also integrates some of the naturally occurring STING antagonists in viruses or humans. In the future, it may be feasible to explore the extraction or synthesis of these antagonists, as well as the development of inhibitors derived from dietary sources. This approach may potentially address the challenges related to the suboptimal pharmacokinetics, physicochemical properties, and side effects associated with small-molecule inhibitors. Moreover, targeted drug delivery systems can be designed to enhance the stability of STING inhibitors and increase the precision of their delivery. Analysing the relationship between STING and metabolic diseases from the perspective of molecular immunology can help comprehensively explore the therapeutic potential of STING targeting in metabolic diseases.

## Figures and Tables

**Table 1:** Mechanism and physiological effects of cGAS inhibitors

Target	Inhibitor	IC50 (μM)	Mechanism	Physiological Effects	Ref.
**cGAS**	**PF-06928215**	4.90	Competes with cGAMP for the active binding checkpoint of cGAS.	No relevant evidence	134
	**RU.521**	0.70	Prevents ATP and GTP from binding to the active site of the enzyme. Benzimidazole-pyrazole/benzothiazole-pyrazole and phthalide moiety in fractions of RU.365 and RU.332 overlap with the adenine ring of ATP and the α/β phosphate of GTP, respectively giving them greater affinity for cGAS than cGAMP.	Effective and selective inhibition of cGAS activity in RAW macrophages and BMDM cells from a mouse model of Aicardi-Goutières syndrome. Reduced expression of IFNB1 in BMDM of in Trex1^-/-^ mouse model of Aicardi-Goutières Syndrome (AGS).	135
	**RU.365**	4.89		Inhibition of IFN-β activation in dsDNA-induced macrophages	
	**RU.332**	13.60			
	**Compound 18**	29.88 ± 3.20	Binds to cGAS via hydrogen bonds with R376 and N482, water-bridge hydrogen bonds with S380 and π-π interactions with Y436.	No relevant evidence	136
	**Compound S3**	4.9 ± 0.26			
	**Compound S2**	13.1 ± 0.09			
	**HYDROXYCHLOROQUINE(HCQ)**	25.0	Block the binding of cGAS to DNA, HCQ occupies a DNA-binding site and is present in the minor groove of the DNA between the protein‒DNA interface of a higher-order catalytically active complex of 2:2 cGAS/DNA.	Inhibition of IFN-β and IL-1β expression in THP1 cells and partially reducing the pyroptosis rate	137
	**QUINACRINE(QC)**	3.00			
	**J001**	hGAS: 1.04 mGAS: 0.490	Prevent ATP and GTP from binding to the active site of the enzyme.	Inhibition of IFN-β expression in human TPH1 cells and mouse RAW264.7 macrophages	138
	**J014**	hGAS: 0.10 mGAS: 0.06			
	**G001, G022, G086, G092, G097, G098**	G001 hGAS: 2.08 mGAS: 0.44	Prevent ATP and GTP from binding to the active site of the enzyme.	Inhibition of IFN-β expression in RAW264 macrophages7	138
	**G108, G150, G140**	G108 hGAS: 0.0275 mGAS: 5.15 G140 hGAS: 0.140 mGAS: 0.442	Occupy an h-cGAS catalytic binding pocket similar to that of GTP and ATP, forming hydrogen bonds with cGAS or inserting the aromatic element of the ligand between Arg376 and Tyr436.	Inhibition of cGAS-STING signalling pathway in human TPH1 cells and primary human macrophages	138
	**Suramin**	N.A.	Acts as a nucleic acid mimic competing with DNA or RNA for the DNA-binding site of cGAS	Reduced IFN-β production induced by dsDNA stimulation of THP1-Dual™ cells	139
	**Aspirin**	N.A.	Prevents binding to DNA by acetylating cGAS at K384 and/or K394 and K414	Inhibition of IRF3 phosphorylation induced by the DNA virus HSV-1. Inhibition of IRF-3-dependent interferon-stimulated genes (ISGs) expression in bone marrow cells of the Trex1 ^-/-^ mouse model of Aicardi-Goutières Syndrome (AGS) Reduction of ISG levels in peripheral blood mononuclear cells (PBMCs) of patients with AGS Alleviation of asymptomatic orchitis in mice due to airborne particulate matter (PM) exposure	140-142
	**CU-32**	0.66±0.10	Block dimerization of cGAS	Inhibition of Sendai virus (SeV)-induced IRF3 activation in human monocytic THP-1 cells	143
	**CU-76**	0.27±0.06			

**Table 2:** Mechanism and physiological effects of STING inhibitors

Target	Type	Compound	Mechanism	Physiological Effects	REF.
**STING**	**Palmitoylation inhibitors**	**H-151**	Bind covalently to the Cys91 site of STING and prevent palmitoylation of STING	Improve cardiomyocyte hypertrophy and reduce collagen deposition in the infarct zone in MI due to reperfusion/myocardial ischaemia. attenuate fibrosis of cardiomyocytes and cardiac fibroblasts in infarction cardiac tissue, relieve the neuropathic pain caused by protein tyrosine phosphate receptor type D (PTPRD) knockout. inhibit the proliferation of diffuse large B-cell lymphoma (DLBCL) tumour cells to promote their apoptosis	31, 144-147
		**C-178**		suppress STING-mediated autoimmune disease by inhibiting interferon-stimulated gene expression in Trex1^-/-^ mouse model of Aicardi-Goutières Syndrome (AGS)	144
		**C-176**		inhibit STING-mediated inflammation-triggered cardiac apoptosis and fibrosis in obesity-associated diabetic cardiomyopathy mice. suppress cardiac inflammation in Trex1^-/-^ mouse model attenuate intestinal ischaemia‒reperfusion-induced lung injury (ALI) and pulmonary fibrosis-induced apoptosis.	144, 148, 149
		**C-170**		inhibit IFN-β and TNF-α expression in TPH1 cells	144
		**C-171**			
	**Nitrofuran derivatives**	**Nitro fatty acids**	Inhibit the palmitoylation of STING.	reduce high-fat diet-induced weight and fatty liver accumulation Inhibit the activation of STING mutants in SAVI-derived fibroblasts and inhibits the release of type I interferon	150-152
		**Astin C**	Blocks the recruitment of STING to IRF3.	reduces inflammatory response in macrophages of Trex1^-/-^ mouse model reduces production of IFN-β cells in MEF cells thereby reducing host resistance to HSV-1 and *Listeria monocytogenes* infection	153
	**Unclear** **mechanism**	**VPX protein**	Inhibits the activity of the STING structural domain that activates NF-κB pathway activity	selectively inhibits NF-κB signalling pathway in HEK293T cells inhibits ISD-stimulated interferon gene expression in EA.hy926 cells (human umbilical vein endothelial cells) inhibits STING-triggered NF-κB signalling in human primary monocyte-derived macrophages and CD4 T cells blocks STING-mediated NF-κB signalling in mice inhibits STING-triggered NF-κB signalling in CD4 Jurkat T cells	154
		**miR-181a**	Targets and binds STING and downregulates STING expression.	blocks cancer cell death and increase immune surveillance in high-grade serous ovarian carcinoma (HGSOC)	155
		**SN-011** (IC_50_: MEFs=127.5±5.5 nM; BMDMs=107.1±16.4 nM; HFFs=502.8±47.3 nM)	Interacts with STING via residues Tyr167, Leu212, Ser243, Tyr245 and Glu260 of STING leads to the occupation of the binding pocket of STING, creating a spatial block and inhibiting the binding of CDN to STING	inhibits overexpression of STING, IRF3 or TBK-1 in HSV-1-infected HFF and 293T cells by phosphorylation and dimerization of signals downstream of STING reduces expression of IFN-β in HEK293 cells inhibits STING activation in patients with SAVI caused by STING or STING mutations attenuates systemic inflammation and reduces the number of activated CD69 CD8 T cells in Trex1^-/-^ mice	156
		**Compound18** (IC_50_=68 nM); **Compound13** (IC_50_=84 nM)	prevent CDNs from attaching to STING by forming a 2:1 binding interaction with the open conformation of STING (PDB ID: 6MX0)	no relevant evidence	157
		**ISD017**	targets the ER Ca2+ sensor stromal interaction molecule 1 (STIM1) to block the release of STING from the endoplasmic reticulum STIMI and its translocation to the Golgi apparatus	blocks STING activity in lupus mouse models and peripheral blood cells (PBMC) of lupus patients	158
		**Viral Protease 3CL&Auxiliary proteins ORF3a**	ORF3a binds to the C- and N-terminal ends of STING to inhibit NF-κB activation and blocks the ability of STING-induced autophagy. 3CL inhibits STING ubiquitination to block TBK1 activation	inhibit STING-mediated expression of NF-κB and IFN-β in HEK293T cells transfected with STING from different species (human, mouse and chicken).	159
		**Palbociclib**	targets STING tyrosine 167 to eliminate the formation of STING dimers	alleviates DSS-induced colitis and attenuate tissue damage due to STING-mediated inflammation in Trex1^-/-^ mouse model	160
		**SP23**	C-170 targets and binds to STING at one end, and the CBRN E3 ubiquitin ligase linked to C-170 degrades STING via the ubiquitin‒proteasome pathway	reduces cGAMP-triggered IFN-β levels in THP-1 cells in a dose-dependent manner attenuates cisplatin-induced acute kidney injury (AKI) by inhibiting STING-mediated inflammation	161
		**UNC93B1**	targets STING degradation via the autophagy‒lysosome pathway	knockdown of UNC93B1 in HEK293T cells and BJ cells enhances the expression of the STING signalling pathway. increases the stability of TBK1 phosphorylation and STING, increases the expression of IFN-β and reduces mortality from HSV-1 infection in UCN92B1^-/-^ mice	162

**Table 3:** Mechanism and physiological effects of TBK1 inhibitors

Target	Inhibitor	Mechanism	Physiological Effects	Ref.
**TBK1**	**Amlexanox**	Competes with ATP for the binding site of the enzyme. Increases phosphorylation of TBK1 at Ser172 in 3T3-L1 adipocytes and blocks poly (I:C)-stimulated phosphorylation of interferon-responsive factor-3 (IRF3)	Increases HFD-induced weight gain and reduces insulin resistance, steatosis and the inflammatory response in DIO mice. Decreases high blood glucose levels and HbA1c levels in a subset of patients with Type 2 diabetes. Attenuates PA-induced inflammation and lipotoxic cell death in hepatocytes and Kupffer cells *in vitro* via inhibition of TBK1/IKKε-NF-κB and/or IRF3. Inhibits CCL4-induced activation and promotion of apoptosis in hepatic stellate cells from mice with liver fibrosis. Inhibits STING/TBK1/IRF3 signalling pathway-mediated allergic asthma development. Promotes lipolysis by inhibiting the activation of PDE3B and enhancing catecholamine sensitivity. Lowers LDL cholesterol to attenuate dyslipidaemia, reduces inflammatory cell infiltration in the aortic vasculature, and reduces monocyte-endothelial cell adhesion to increase aortic vascular cell dysfunction. Increases cardiac function and reduces myocardial inflammatory response and myocardial apoptosis in post-AMI rats.	163
	**GSK8612**	Establishes two conserved hydrogen bonding interactions with the hinge region of the kinase backbone in IRF3.	Inhibits the phosphorylation of IRF3 in Ramos cells and IFN-β secretion in human THP-1 cells.	163
	**BX795** (IC_50_=0.006 ± 0.001 μM);	Phosphorylates TBK1 and IKKϵ at Ser-172	Increases the phosphorylation level of TBK1 approximately 2-fold in LPS-stimulated RAW264.7 and primary bone marrow-derived macrophages.	164
	**Compound 7 l** (IC_50_= 22.4 nM)	The aminopyrrolo [2,3-d] pyrimidine core forms a divalent hydrogen bond with Cys89 of TBK1, the cyclobutene carboxamide side chain forms a water-mediated hydrogen network with Lys38 of TBK1, and the acetyl piperazine hydrophilic group extends into the solvent region by forming a direct hydrogen bond with Phe90 of TBK1.	Inhibit IFN-β and CXCL7 expression in LPS-stimulated human THP1-Blue-ISGs cells and murine RAW264.7 cells. Moderately inhibit the proliferation of A549 lung cancer cells (IC50=17.6μM) and mouse Lewis lung cancer (LLC) cells (IC50=9.4μM).	165
	**BAY-985**	binds to the hinge region within the ATP site of the kinase domain	Weakly suppresses tumour in SK-MEL-2 human melanoma xenograft model.	166
	**PROTAC 3i**	Compound 3i binds to TBK1 and the VHL E3 ubiquitin ligase linked to Compound 3i to degrade TBK1 via the ubiquitin‒proteasome pathway.	Nearly totally degrades TBK1 in K-Ras mutant cell lines (H23, A549 and H1792).	167
	**pS273R**	Inhibits IKKε interaction with STING by targeting IKKε to block its SUMOylation.	Disrupts cGAS-STING signalling-mediated anti-VSV function.	168

## Abbreviations

AAD: aortic aneurysm and dissection
AKT: protein kinase B
AMPK: AMP-activated protein kinase
ATKO: adipocyte-specific TBK1 knockout
ATP: adenosine triphosphate
BMDM: bone-marrow-differentiated macrophages
CDNs: cyclic dinucleotides
cGAMP: 2':3'-cyclic GMP-AMP
cGAS: cyclic GMP-AMP synthase
CTT: C-terminal tail
CVDs: cardiovascular diseases
Cys: cysteine
DsbA-L: disulfide-bond A oxidoreductase-like protein
dsDNA: double-stranded DNA
ER: endoplasmic reticulum
GLUT4: glucose transporter 4
GSDMD: Gasdermin D
GTP: guanosine triphosphate
HFD: high-fat diet
HSCs: hepatic stellate cells
ICAM-1: intracellular adhesion molecule 1
IFN-β: interferon β
IFNAR: interferon receptors
IKK: IkB kinase
IL: interleukin
IRF3: interferon regulatory factor 3
IR: insulin receptor
IRS1: insulin receptor substrate 1
ISGs: IRF-3-dependent interferon-stimulated genes
JNK: Jun N-terminal kinase
KCs: Kupffer cells (KCs)
LBD: ligand-binding domain
LC3: light chain 3
LPS: lipopolysaccharide
MCP-1: monocyte chemoattractant protein-1
MI: myocardial infarction
MST1: mammalian Ste20-like kinases 1
mtDNA: mitochondrial DNA
mTOR: mammalian target of rapamycin
MyD88: myeloid differentiation factor 88
NAFLD: nonalcoholic fatty liver disease
NASH: nonalcoholic steatohepatitis
NF-κB: nuclear factor-kappa B
NLRP3: NOD-: LRR- and pyrin domain-containing protein 3
PDE3B: phosphodiesterase-3B
PKA: protein kinase A
Poly(I:C): polyinosinic-polycytidylic acid
PRRs: pattern recognition receptors
SASP: senescence-associated secretion profile
SAVI: STING-associated vasculopathy with onset in infancy
Ser: serine
SMC: smooth muscle cell
SNPs: single nucleotide polymorphisms
STIM1: stromal interaction molecule 1
STING: stimulator of interferon genes
TBK1: TANK-binding kinase 1
TLR: Toll-like receptor
TM: transmembrane
TNF-α: tumour necrosis factor-alpha
TREX1: three prime repair exonuclease 1
TRIF: TIR domain-containing adaptor inducing interferon-β
ULK1: Unc-51-like protein kinase 1
WAT: white adipose tissue
YAP: Yes-associated protein
